# Differences in Aortic Histopathology in Patients Undergoing Valve Reimplantation Surgery for Various Clinical Syndromes

**DOI:** 10.1055/s-0042-1743536

**Published:** 2022-08-07

**Authors:** Nicholas J. Tucker, Tal Eitan, Justin G. Yoon, Bradley F. Rosinski, E. Rene Rodriguez, Carmela D. Tan, Lars G. Svensson

**Affiliations:** 1 Case Western Reserve University School of Medicine, Cleveland, Ohio; 2 Department of Thoracic and Cardiovascular Surgery, Cleveland Clinic, Cleveland, Ohio; 3 Department of Pathology, Cleveland Clinic, Cleveland, Ohio

**Keywords:** aortic histopathology, aortic valve reimplantation, bicuspid aortic valve, connective tissue disease

## Abstract

**Objectives** The study aims to investigate aortic histopathologic differences among patients undergoing aortic valve reimplantation, suggest different mechanisms of aortic root aneurysm pathogenesis, and identify factors associated with long-term success of reimplantation.

**Methods** From 2006 to 2017, 568 adults who underwent reimplantation for repair of aortic root aneurysm, including patients with tricuspid aortic valves with no connective tissue disease (TAV/NoCTD, *n* = 314/568; 55.3%), bicuspid aortic valves (BAVs, *n* = 86/568; 15.1%), or connective tissue disease (CTD, *n* = 177/568; 31.2%), were compiled into three comparison groups. Patients with both BAV and CTD ( *n* = 9/568; 1.6%) were omitted to increase study power. Patient records were analyzed retrospectively, focusing on pathology reports, which were available for 98.42% of patients, and were classified based on their descriptions of aortic tissue samples, primarily from the noncoronary sinus. Mean follow-up time available for patients was 2.97 years.

**Results** Aortitis, medial fibrosis, and smooth muscle loss were more common histopathologic findings in patients with TAV/NoCTD than in patients with BAV and CTD ( *p* < 0.05). Cystic medial degeneration was most often found in patients with CTD, then TAV/NoCTD, and least in BAV ( *p* < 0.01). Increases in mucopolysaccharides were found more often in the BAV group than in the TAV/NoCTD and CTD groups ( *p* < 0.01). There were no differences in the frequency of elastic laminae fragmentation/loss across these three groups. Among all patients, 1.97% ( *n* = 11/559) had an unplanned reintervention on the aortic valve after reimplantation, but no significant demographic or histopathologic differences were identified.

**Conclusion** Despite some common histopathologic features among patients undergoing aortic valve reimplantation, there were enough distinguishing features among aortic tissue samples of TAV/NoCTD, BAV, and CTD patients to suggest that these groups develop root aneurysms by different mechanisms. No histopathologic features were able to predict the need for late reintervention on the aortic valve.

## Introduction

For patients with bicuspid aortic valves (BAV) and connective tissue disease (CTD), including Marfan syndrome, Ehlers-Danlos syndrome, and Loeys-Dietz syndrome, a well-documented association with aneurysms, dissections, and ruptures of the thoracic aorta is of particular concern. More than 35% of patients with BAV will require interventions due to complications of the aortic valve and ascending aorta, and dissection and rupture of the aortic root are the leading cause of death among patients with Marfan syndrome. 1 2 Despite the clinical significance of this association, the exact mechanism by which these conditions lead to thoracic aortic complications is not yet fully elucidated. 1 Filling the gaps in this knowledge may not only provide insight into the pathogenesis of aortic dilatation in the setting of CTD, but also may suggest strategies for predicting and managing the course of disease.

In this study, we examined the histopathologic findings of 559 patients who underwent aortic valve reimplantation from 2006 to 2017. By comparing these findings between patients with BAV, CTD, and those without either, we can suggest different mechanisms for the development of aortic root aneurysms and seek to identify factors associated with the long-term success of aortic valve reimplantation.

## Materials and Methods

Between January 2006 and March 2017, 568 patients underwent aortic valve reimplantation for repair of aortic root aneurysm, including patients with tricuspid aortic valves with no CTD (TAV/NoCTD, *n* = 314/568; 55%), BAVs ( *n* = 86/568; 15%), or CTD ( *n* = 177/568; 31%). Patients who were documented to have both a BAV and a CTD accounted for only 1.6% of the study sample ( *n* = 9/568) and were consequently omitted from some analyses, as further described below. All consecutive patients who received the reimplantation procedure during the above time period were included in the study with informed consent waived, as approved by the institutional review board. Data collection consisted of retrospective review of patients' medical records, imaging studies, pathology studies, and operative reports. The mean follow-up time among all patients was 2.97 years.

**Fig. 1:**
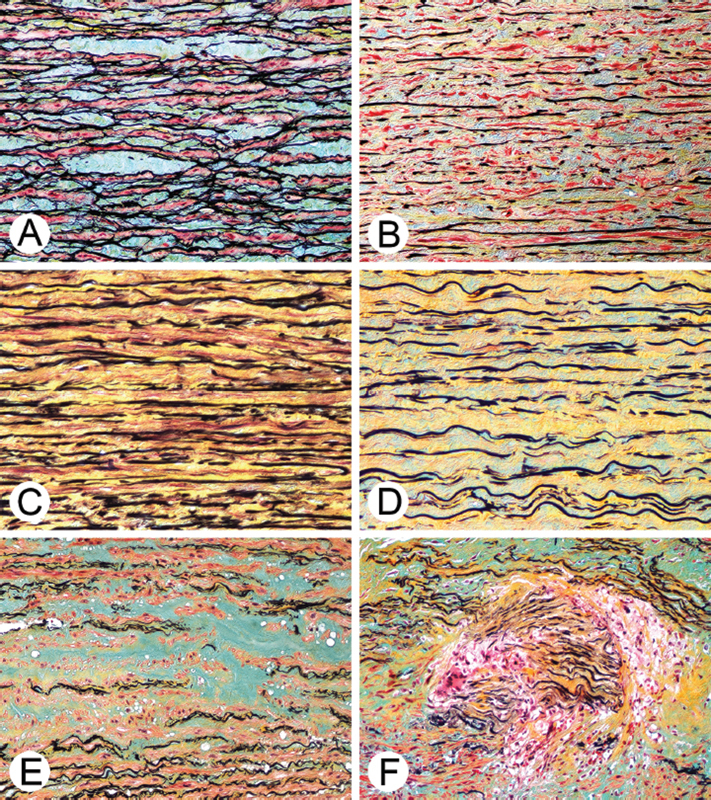
Pathology slides of the six major histopathologic categories analyzed according to the *Consensus Statement on Surgical Pathology of the Aorta from the Society for Cardiovascular Pathology and the Association for European Cardiovascular Pathology: II* . Histopathology of the aorta is shown using Movat pentachrome stain in which the smooth muscle cell cytoplasm is stained red, elastic lamellae black, mucopolysaccharide matrix blue-green, and collagen yellow. ( **A** ) Mucopolysaccharide accumulation within elastic lamellar units. ( **B** ) Elastic lamellar fragmentation. ( **C** ) Medial fibrosis without smooth muscle cell loss. ( **D** ) Medial fibrosis with smooth muscle cell loss. ( **E** ) Cystic medial degeneration with elastic lamellar loss and marked accumulation of mucopolysaccharides. ( **F** ) Aortitis with multinucleated giant cells.

All pathology reports that were available from the Epic electronic health record and corresponded to a patient's reimplantation procedure were included in the study, including reports for 98.42% ( *n* = 559/568) of patients. All reports were generated from two staff pathologists at Cleveland Clinic, who were the sole cardiovascular pathologists for the institution throughout the period of study, with histopathologic findings based on tissue samples collected during surgery, most often from the noncoronary sinus of a patient's aorta. All reported findings were tabulated into different description groups based on specific phrases used by the pathologists, including findings such as “aortitis,” “cystic medial degeneration,” “elastic laminae fragmentation,” etc. All significant phrases from patients' pathology reports were included in initial analysis and then were ultimately placed into appropriate categories in accordance with the *Consensus Statement on Surgical Pathology of the Aorta from the Society for Cardiovascular Pathology and the Association for European Cardiovascular Pathology: II* . 3 Ultimately, the six histopathologic categories used for analysis included aortitis, cystic medial degeneration, elastic lamellar fragmentation and loss without overt cystic medical degeneration, medial fibrosis without smooth muscle cell loss, mucopolysaccharide accumulation/increase without overt cystic medial degeneration, and smooth muscle cell loss/laminar necrosis. For simplification of communication, these categories will be referred to throughout this study as “aortitis,” “cystic medial degeneration,” “elastic laminae fragmentation/loss,” “medial fibrosis,” “mucopolysaccharide increase,” and “smooth muscle cell loss” ( Fig. 1 ).

### Surgical Techniques

Overall, the vast majority of operations were performed on an elective basis ( *n* = 544/568, 95.77%), with the remaining operations considered either emergency ( *n* = 16/568, 2.82%) or urgent ( *n* = 8/568, 1.41%). Major clinical indications for operation on these patients included aortic root aneurysm and aortic insufficiency; however, Type A dissection was listed as a surgical indication for 15 patients (2.64%), and Type B dissection was a comorbid condition for 12 patients (2.11%).

Among all operations, redo sternotomies were performed on 27 patients (4.75%). Concomitant operations included aortic valve repair ( *n* = 204/568, 35.92%), mitral valve repair ( *n* = 46/568, 8.10%), tricuspid valve repair ( *n* = 12/568, 2.11%), coronary artery bypass grafting ( *n* = 43/568, 7.57%), total aortic arch replacement ( *n* = 28/568, 4.93%), hemiarch replacement ( *n* = 53/568, 9.33%), conventional elephant trunk procedure ( *n* = 15/568, 2.64%), and frozen elephant trunk procedure ( *n* = 12/568, 2.11%). The figure-of-8 suturing technique was used for aortic valve repair 31.37% of the time ( *n* = 64/204).

### Statistical Analysis

Statistical analysis was performed using Microsoft Excel 2016 and R statistical software. Comparisons among the three main patient groups (TAV/NoCTD, BAV, CTD) were conducted primarily through chi-square analyses and analysis of variance with a significance value of 0.05, unless otherwise specified. To accurately meet this *p* -value requirement of 0.05, a Bonferroni correction was applied for the six major histopathologic groupings, making the level of significance for those comparisons *p* < 0.0083. A *p* -value requirement of 0.01 after Bonferroni correction would require *p* < 0.0017. Post hoc analyses were then conducted by chi-square analyses and two-tailed unequal variance Student's *t* -tests as appropriate. As noted earlier, the group with coexisting BAV and CTD was excluded from these particular tests to avoid reduction in statistical power because of its small sample size ( *n* = 9).

Analyses related to late reintervention on the aortic valve were also conducted, with comparisons being made between patients requiring some type of aortic valve reintervention (AVReInt, *n* = 11/559, 1.97%) and patients not requiring a reintervention on the aortic valve (NoReInt, *n* = 548/559, 98.03%). The group of nine patients with coexisting BAV and CTD was excluded from analysis. Tests conducted for these analyses included chi-square analyses and two-tailed unequal variance Student's *t* -tests with a significance value of 0.05, unless otherwise specified.

## Results

### Patient Demographics

Basic patient demographics, including age, sex, and comorbidities among the three main groups of comparison (TAV/NoCTD, BAV, CTD), can be found in Table 1 . Significant differences were found for age and sex, as well as comorbidities of coronary artery disease, prior myocardial infarction, prior stroke, hypertension, hyperlipidemia, and any type of cancer—and all of these differences remained significant at a *p* -value level of 0.01 after Bonferroni correction ( *p* < 0.0007 for the 15 comparisons made) except for prior myocardial infarction ( *p* = 0.011) and prior stroke ( *p* = 0.028).

**Table 1:** Basic demographics of patient population, tricuspid aortic valves with no connective tissue disease versus bicuspid aortic valves versus connective tissue disease

	TAV/NoCTD	BAV	CTD	*p* -Value
	*n* = 314 (%)	*n* = 77 (%)	*n* = 168 (%)	
Age	51.9 (range: 16–79)	46.2 (range: 17–70)	37.6 (range: 13–73)	** < 0.01 b**
Male/Female	272 (86.6) / 42 (13.4)	69 (89.6) / 8(10.4)	119 (70.8) / 49 (29.2)	** < 0.01 b**
Smoking	140 (44.6)	24 (31.2)	63 (37.5)	0.06
*Comorbidities* :				
Congestive heart failure	23 (7.3)	5 (6.5)	5 (3.0)	0.15
Coronary artery disease	114 (36.3)	13 (16.9)	25 (14.9)	** < 0.01 b**
Prior myocardial infarction	17 (5.4)	1 (1.3)	1 (0.6)	** < 0.05 a**
Prior stroke	13 (4.1)	2 (2.6)	0 (0.0)	** < 0.05 a**
Hypertension	221 (70.4)	53 (68.8)	89 (53.0)	** < 0.01 b**
Diabetes mellitus	15 (4.8)	1 (1.3)	9 (5.4)	0.33
Hyperlipidemia	162 (51.6)	29 (37.7)	43 (25.6)	** < 0.01 b**
Chronic obstructive pulmonary disease	17 (5.4)	1 (1.3)	8 (4.8)	0.31
Chronic kidney disease	10 (3.2)	1 (1.3)	1 (0.6)	0.15
Cancer (any)	36 (11.5)	0 (0)	6 (3.6)	** < 0.01 b**

Abbreviations: BAV, bicuspid aortic valves; CTD, connective tissue disease; TAV/NoCTD, tricuspid aortic valves with no connective tissue disease.

a Indicates that the comparison demonstrated significance ( *p* < 0.05) after applying a Bonferroni correction.

b Indicates that the comparison demonstrated significance ( *p* < 0.01) after applying a Bonferroni correction.

### Aortic Histopathology

Initial chi-square analyses of the pathology reports among the three main groups of comparison (TAV/NoCTD, BAV, and CTD) revealed that there was a significant difference between these groups related to the histopathologic parameters of “aortitis” ( *p* < 0.01), “cystic medial degeneration” ( *p* < 0.01), “medial fibrosis” ( *p* < 0.01), “mucopolysaccharide increase” ( *p* < 0.01), and “smooth muscle cell loss” ( *p* < 0.01). No difference was found between these groups for “elastic laminae fragmentation/loss” ( *p* = 0.75).

**Fig. 2:**
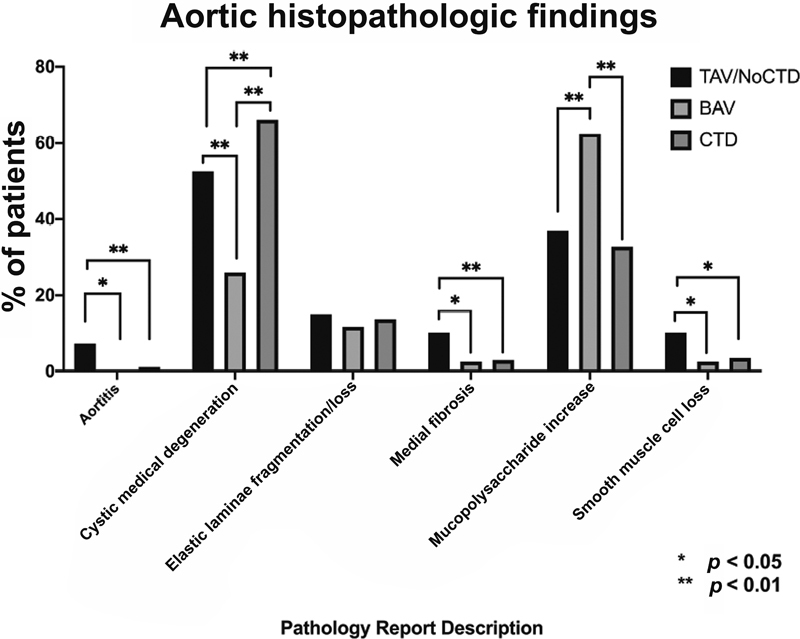
Aortic histopathologic findings among tricuspid aortic valve with no connective tissue disease (TAV/NoCTD), bicuspid aortic valve (BAV), and connective tissue disease (CTD).

In applying a Bonferroni correction (at a level of *p* < 0.05 and *p* < 0.01) to all of these findings for the six main histopathologic comparisons made, the *p* -value threshold for significance was adjusted to *p* < 0.0083 and *p* < 0.0017, respectively. In meeting these correction requirements, the findings for “medial fibrosis” and “mucopolysaccharide increase” met the *p* < 0.0083 threshold, while the findings for “aortitis,” “cystic medial degeneration,” and “smooth muscle cell loss” met the *p* < 0.0017 threshold. These findings are all summarized in Table 2 . Ensuing post hoc analyses are presented in Fig. 2 and will be described in the paragraphs below.

**Table 2:** Aortic histopathology findings, tricuspid aortic valves with no connective tissue disease versus bicuspid aortic valves versus connective tissue disease

	TAV/NoCTD	BAV	CTD	*p* -Value
	*n* = 314 (%)	*n* = 77 (%)	*n* = 168 (%)	
Aortitis	23 (7.3)	0 (0.0)	2 (1.2)	** < 0.01 b**
Cystic medial degeneration	165 (52.5)	20 (26.0)	111 (66.1)	** < 0.01 b**
Elastic laminae fragmentation/loss	47 (15.0)	9 (11.7)	23 (13.7)	0.75
Medial fibrosis	32 (10.2)	2 (2.6)	5 (3.0)	** < 0.01 a**
Mucopolysaccharide increase	116 (36.9)	48 (62.3)	55 (32.7)	** < 0.01 a**
Smooth muscle cell loss	32 (10.2)	2 (2.6)	6 (3.6)	** < 0.01 b**

a Indicates that the comparison demonstrated significance ( *p* < 0.05) after applying a Bonferroni correction.

b Indicates that the comparison demonstrated significance ( *p* < 0.01) after applying a Bonferroni correction.

Post hoc analyses demonstrated that the TAV/NoCTD group had a higher prevalence of “aortitis” (7.3%), as compared with both the BAV (0%, *p* < 0.05) and CTD (1.2%, *p* < 0.01) groups. Additionally, the TAV/NoCTD group had a higher prevalence of “medial fibrosis” (10.2%) as compared with the BAV (2.6%, *p* < 0.05) and CTD (3.0%, *p* < 0.01) groups, as well as a higher prevalence of “smooth muscle cell loss” (10.2%), as compared with the BAV (2.6%, *p* < 0.05) and CTD (3.6%, *p* < 0.05) groups.

The BAV group was noted to have the highest prevalence of “mucopolysaccharide increase” (62.3%), being significantly higher than both the TAV/NoCTD (36.9%, *p* < 0.01) and CTD groups (32.7%, *p* < 0.01).

**Fig. 3:**
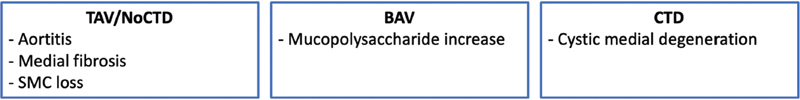
Simplified schematic demonstrating the main aortic histopathologic findings associated with tricuspid aortic valve with no connective tissue disease (TAV/NoCTD), bicuspid aortic valve (BAV), and connective tissue disease (CTD) after analysis.

Finally, prevalence of “cystic medial degeneration” was greatest in patients with CTD (66.1%), then TAV/NoCTD (52.5%), and least in BAV (26.0%, *p* ≤ 0.01 for all comparisons). These major histopathologic findings are presented with their associated conditions using a simplified schematic in Fig. 3 .

### Morbidity and Mortality

In examining safety of reimplantation operations overall, death within 30 days of operation occurred in only 3 patients (0.53%). Regarding complications, operative stroke occurred in 8 patients (1.41%), operative paralysis occurred in 5 patients (0.88%), operative paresthesia occurred in 12 patients (2.11%), myocardial infarction occurred in 7 patients (1.23%), and reoperations for bleeding were performed in 40 patients (7.04%).

### Aortic Valve Reinterventions

In our sampled population, 1.97% of patients ( *n* = 11/559) had an unplanned reintervention on the aortic valve postreimplantation. As summarized in Table 3 , there were no significant differences in age, sex, smoking status, or comorbidities between the patients who underwent late aortic valve reintervention and those who did not.

**Table 3:** Basic demographics of patient population, aortic valve (AV) reintervention versus no AV reintervention

	AV reintervention	No AV reintervention	*p* -Value
	*n* = 11 (%)	*n* = 548 (%)	
Age	38.3	47.0	0.07
Male/Female	10 (90.9)/1 (9.1)	450 (82.1)/98 (17.9)	0.45
Smoking	6 (54.5)	221 (40.3)	0.34
*Comorbidities* :			
Bicuspid aortic valve	3 (27.3)	74 (13.5)	0.19
Connective tissue disease	2 (18.2)	166 (30.3)	0.39
Congestive heart failure	1 (9.1)	32 (5.8)	0.65
Coronary artery disease	3 (27.3)	149 (27.2)	1.00
Prior myocardial infarction	0 (0.0)	19 (3.5)	0.53
Prior stroke	1 (9.1)	14 (2.6)	0.18
Hypertension	5 (45.5)	358 (65.3)	0.17
Diabetes mellitus	0 (0.0)	25 (4.6)	0.47
Hyperlipidemia	3 (27.3)	231 (42.2)	0.32
Chronic obstructive pulmonary disease	0 (0.0)	26 (4.7)	0.46
Chronic kidney disease	0 (0.0)	12 (2.2)	0.62
Cancer (any type)	0 (0.0)	42 (7.7)	0.34

When analyzing for aortic histopathologic differences between these two groups, there were no significant differences for any of the six categories of “aortitis” ( *p* = 0.47), “cystic medial degeneration” ( *p* = 0.27), “elastic laminae fragmentation/loss” ( *p* = 0.63), “medial fibrosis” ( *p* = 0.78), “mucopolysaccharide increase” ( *p* = 0.67), or “smooth muscle cell loss” ( *p* = 0.35) in relation to aortic valve reinterventions. A summary of these histopathologic findings can be found in Table 4 .

**Table 4:** Aortic histopathology findings, aortic valve (AV) reintervention versus no AV reintervention

	AV reintervention	No AV reintervention	*p* -Value
	*n* = 11 (%)	*n* = 548 (%)	
Aortitis	0 (0.0)	25 (4.6)	0.47
Cystic medial degeneration	4 (36.4)	292 (53.3)	0.27
Elastic laminae fragmentation/loss	1 (9.1)	78 (14.2)	0.63
Medial fibrosis	1 (9.1)	38 (6.9)	0.78
Mucopolysaccharide increase	5 (45.5)	214 (39.1)	0.67
Smooth muscle cell loss	0 (0.0)	40 (7.3)	0.35

## Discussion

Normal histology of the ascending aorta reveals three layers: the tunica intima, tunica media, and tunica adventitia, from the inside (luminal aspect) to the outside of the vessel, respectively. The tunica media layer, the largest component of the aortic wall, is composed of 60 to 70 layers of lamellar units, which are composed of smooth muscle cells, collagen, and proteoglycans, between two sheets of elastic lamellae. 3 Disruption of lamellar units is routinely evaluated during pathologic examination, and aortic aneurysms and dissections are specifically associated with medial degeneration. 4 Due to the potentially fatal outcomes of these conditions, it is imperative to investigate the components of the medial layer of the aorta to understand the pathogenesis of the degeneration.

Several studies have investigated the role of mucopolysaccharides, also known as proteoglycans, in both the nonpathologic and pathologic states of the aorta. In the nonpathologic state, for example, Azeloglu et al 5 verified their hypothesis that proteoglycans produce a Donnan osmotic pressure, helping to provide residual stress to the aorta to allow for homeostatic maintenance of uniform distribution of wall stress. Increasing the proteoglycan production, degradation, and distribution is how vascular cells respond to changes in wall stress from blood pressure or flow. 5 The negative charges on glycosaminoglycan side chains attract mobile counterions, such as sodium; water then follows the sodium, creating pressure in the region. 6 This swelling changes the tension on the connections between the microfibrils and the smooth muscle cells, facilitating the mechanosensory function of smooth muscle cells. 6

However, if proteoglycan levels accumulate to pathologic levels, they can generate an osmotic pressure that can disrupt the extracellular matrix within the lamellar unit. 6 This disruption has biochemical, electrochemical, and mechanobiological ramifications. 7 Specifically, Cikach et al 6 have discussed the characteristics of different proteoglycans, aggrecan and versican, that belong to the proteoglycanomes of both nonpathologic and pathologic states of the aorta. For example, glycosylated versican demonstrates antiadhesive properties. 6 Smooth muscle cells, endothelial cells, fibroblasts, and cardiac myocytes require adhesion to structural glycoproteins for survival—and adhesion represses apoptotic signals and allows for tensional integrity. 8 Therefore, antiadhesive properties play a role in the deterioration of smooth muscle cells. 6 Furthermore, Michel 8 discussed how a loss of interactions between cells and the extracellular matrix prompts smooth muscle cell apoptosis by a process known as anoikis. Thus, depending on the degree, proteoglycan accumulation throughout the aorta might lead to pathologic states.

For the case of thoracic aortic aneurysms and dissections (TAADs), Cikach et al 6 used ribonucleic acid in situ hybridization studies to demonstrate a rise in gene expression of aggrecan and versican in humans and in mouse models. They also noted reduced expression of *ADAMTS5* , a protease that contributes to the degradation of proteoglycans. 6 Thus, given increased production and decreased turnover of proteoglycans, proteoglycans are able to accumulate. Cikach et al 6 speculated that the process of proteoglycan accumulation is accelerated in TAAD because of genetic defects in the connections between cells and extracellular matrix of lamellar units. It has been established that proteoglycans are present in states of TAAD. 3 Nonetheless, the role that proteoglycans play in the pathogenesis of TAAD is currently being investigated. 6

Given our observations that the BAV group of aortic root biopsies exhibited an increased frequency of “mucopolysaccharide increase,” we postulate that individuals with BAV might develop aortopathy, such as TAAD, at a faster rate and/or to a greater degree than patients who have a TAV and no coexisting CTD. Greater proteoglycan content or distribution in the aortic wall could result in a greater Donnan osmotic swelling pressure and loss of tensile stiffness, creating a microenvironment that facilitates the development of stress concentrations. 9

In our study, we did not observe any differences in the frequency of elastic laminae fragmentation and loss among patients with TAV/NoCTD, BAV, or CTD. However, several other studies have previously documented that the aortas in patients with BAV tend to have well-organized elastic lamellae that are thinner and have a greater interlamellar distance. 10 11 Some published literature has demonstrated disagreement with this finding, such as a study from de Sa et al 12 that reported that elastic fragmentation, smooth muscle cell changes, and cystic medial degeneration were significantly more severe in patients with BAV as compared with patients with TAV. Other studies have identified possible biomarkers that suggest an alternative mechanism for the development of aneurysm in patients with BAV, including elevated levels of matrix metalloproteinases (MMP). In particular, the turbulent flow and shear stress associated with BAV may activate MMP-2, which is predominantly expressed in the outer curvature of the ascending aorta and cleaves numerous collagen subtypes and fibrillin. On the other hand, elastic fragmentation in TAV may be driven by elevated levels of MMP-13. 13 14 Seeing as we could not distinguish a difference in this parameter, the role of elastic laminae fragmentation and loss remains unclear between these groupings.

The CTD group was noted to have greater amounts of “cystic medial degeneration” than the BAV and TAV/NoCTD groups. This is consistent with multiple studies that have specifically associated Marfan syndrome with more overall medial degeneration. 11 15 However, the graded effect seen with the BAV group having less frequent cystic medial degeneration than the TAV/NoCTD group is somewhat in disagreement with the study from de Sa et al 12 —though this may be more of a difference in sampling error and/or classification between frequency and severity of cystic medial degeneration, as the present study was only designed for frequencies of the pathology phrase.

While we have highlighted some histopathologic differences in this study, there may be some overlap between the mechanism of aortic wall complications between patients with BAV and CTD. In particular, a study by Grewal and Gittenberger-de Groot 16 examined specimens from the aortas of patients with Marfan syndrome and BAV and found that a significant lack of differentiation of vascular smooth muscle cells (VSMCs) appears to play a role in the disease process of both. This was highlighted by a lack of smoothelin, a marker of highly differentiated VSMCs, and localization of fibrillin-1 that was intracellular, as opposed to the extracellular localization observed in aortas with TAV and no CTD. This overlap, they hypothesized, may be attributed to the common embryological origin of the semilunar valves and the VSMCs of the ascending aorta and aortic root, both of which are derived from neural crest cells and second heart field progenitor cells.

Finally, the TAV/NoCTD group was notably distinguished from the BAV and CTD groups by its higher frequencies of “aortitis,” “medial fibrosis,” and “smooth muscle cell loss.” Other preliminary analyses in preparation for this study discovered that common phrases in the “aortitis” group also included “inflammation,” “neointimal hyperplasia,” and “plasma cells.” These phrases all share an inflammatory and/or chronic disease picture of pathogenesis that differs from the more congenital mechanisms at play in BAV and CTD patients. Aortitis, including giant cell arteritis and clinically isolated aortitis, are typically more common in older patients, and present as aortic aneurysms. The findings of “medial fibrosis” and “smooth muscle cell loss” are also intuitively consistent with the demographic findings that the TAV/NoCTD group tended to present for surgery at older ages, and that these findings were at least in part related to age-related changes.

In reintervention analyses, there were no statistically detectable differences in either demographics of histopathology noted between patients who underwent an unplanned reintervention on the aortic valve and those who did not. While this result may simply be due to a lack of power (as this comparison was significantly unbalanced in population sizes), this study's design ultimately could not identify any useful patient characteristics or histopathologic factors in predicting need for a future procedure on the aortic valve.

There were some limitations in this study that may have influenced some of our findings. Broadly speaking, the design does revolve around a single-center, retrospective chart review of many histological descriptions of aortic specimens spanning 11 years of patients. While one concern with this could be that there were variations in phrasing and/or interpretation of findings among pathology readings, this was controlled for by including a range of dates for which there were only two staff cardiovascular pathologists in charge of these reports. Additionally, initial data collection from patient charts were tabulated with any and all descriptions from every pathology report first before being sorted later into their representative categories to best reflect the recommendations based on the *Consensus Statement on Surgical Pathology of the Aorta from the Society for Cardiovascular Pathology and the Association for European Cardiovascular Pathology: II* . 3 Despite these efforts, it is understood that depending on the size and location of a tissue sample that was retrieved from surgery, there may have been differing amounts of findings on the histologic slides, which is a limitation of sampling error that could not be addressed in this retrospective study.

Ultimately, this study was intended as a preliminary investigation into what histopathologies are found in some of the major patient groups that we see presenting with thoracic aortic aneurysms. Because the sample is drawn from consecutive patients, the results of the study are representative of the patients who receive aortic valve reimplantation procedures at our institution. Since there were no patient matching analyses performed, there were a few notable, significant differences in patient demographics. This obviously does not mean that all of our findings can be contributed to our defined groupings alone, but this study does help describe how the groups that we see commonly present, such as how patients with CTD tend to require surgical management at younger ages.

In recognition of these limitations, research on the development of thoracic aortic aneurysms would certainly benefit from more prospective studies that specifically examine the histopathologies of patient tissue samples. Some prospective research has already been done in mouse models, such as in a study by Cikach et al, 6 which observed increased aggrecan staining in mice with Marfan syndrome; similar, ongoing research at Cleveland Clinic using human aortic specimens will continue to provide insight surrounding our understanding of the differing pathophysiologies of aortic aneurysms.

## Conclusions

This preliminary, single-center, retrospective study was designed to investigate the histopathologic features of patients who present for aortic valve reimplantation procedures to suggest different mechanisms for the pathogenesis of aortic root aneurysms and to identify factors associated with long-term success of aortic valve reimplantation. While there were many common histopathologic features among patients undergoing aortic valve reimplantation, there were enough distinguishing features among aortic tissue samples of TAV/NoCTD, BAV, and CTD patients to propose that these patient groups develop aortic root aneurysms by different mechanisms. However, while these pathology report findings may provide insight into the different ways that aortic root aneurysms develop, there were no significant histopathologic findings that could predict the need for late reintervention on the aortic valve.
